# Xanomeline-Trospium Chloride as a New Paradigm in the Treatment of Schizophrenia Through Muscarinic Modulation: A Renewed Hope in Psychiatric Care

**DOI:** 10.7759/cureus.102250

**Published:** 2026-01-25

**Authors:** Mohsin Raza, Jasleen Kaur

**Affiliations:** 1 Psychiatry, Dover Behavioral Health System, Dover, USA; 2 Psychiatry, University of Connecticut Health, Farmington, USA

**Keywords:** antipsychotics, cobenfy, schizophrenia, trospium chloride, xanomeline, xanomeline-trospium

## Abstract

Schizophrenia remains one of the most challenging clinical issues, despite the efforts made and the number of treatments available to date. This review examines xanomeline-trospium chloride, a novel therapeutic approach to treating schizophrenia by targeting muscarinic acetylcholine receptors (mAChRs). The drug consists of xanomeline, a selective M1/M4 receptor agonist, and trospium, a peripheral muscarinic antagonist, which are used, respectively, to meet central therapeutic needs and avoid peripheral side effects. Xanomeline-trospium chloride has demonstrated clinical efficacy. Moreover, xanomeline-trospium chloride exhibited a better safety profile compared to prior agents, with no major metabolic impact and low discontinuation rates. However, studies show transient mild to moderate gastrointestinal effects.

Nevertheless, the clinical trials of this drug have shown that it is effective and well tolerated. Although xanomeline-trospium chloride is already approved for the treatment of schizophrenia, particularly for patients who need better metabolic and cognitive outcomes, future studies should aim to investigate its efficacy in treatment-resistant schizophrenia, early-stage intervention, and other neuropsychiatric disorders. This review aims to integrate the current evidence and assess the potential of xanomeline-trospium chloride as a novel therapeutic approach in the management of schizophrenia.

## Introduction and background

Schizophrenia is a chronic and severe mental illness that affects about 23 million people worldwide [1]. It imposes a significant burden on patients, families, and society due to the severity of the symptoms and the need for care. The disorder is characterized by disruptions in thoughts, perceptions, emotions, and behavior, which lead to social and occupational dysfunction [2]. Even though various antipsychotic drugs have been developed, the treatment of schizophrenia remains a clinical problem. Many patients have suboptimal symptom control, experience significant side effects, or have a limited response to current pharmacotherapies [3,4].

The pathogenesis of schizophrenia is complex and involves genetic, neurobiological, and environmental factors, which lead to neurochemical imbalance, especially in the dopaminergic, glutamatergic, and cholinergic systems [5,6]. One of the most important areas of interest in the present study of schizophrenia is the cholinergic system and its receptors, which have been shown to play an important role in the pathogenesis of the positive, negative, and cognitive symptoms of the disorder [4,7]. Muscarinic receptors are G-protein-coupled receptors involved in various physiological functions, including cognition, attention, and information processing [4,8]. In schizophrenia, the expression of certain muscarinic receptor subtypes, including the M1 and M4 subtypes, is altered and is associated with the onset and course of the illness [4,6]. Preclinical studies have demonstrated that targeting these muscarinic receptor subtypes affects cognition and modulates both classic positive and negative symptoms [5,9].

Xanomeline-trospium chloride, approved in 2024, is a significant advance in the management of schizophrenia and represents a change in the paradigm of schizophrenia treatment. This novel therapeutic combines xanomeline, a muscarinic acetylcholine receptor (mAChR) agonist targeting the M1 and M4 receptor subtypes, with trospium chloride, a peripherally restricted muscarinic antagonist [10,11]. By selectively activating central M1 and M4 receptors, xanomeline is believed to lower dopamine transmission, while trospium mitigates peripheral cholinergic side effects, such as gastrointestinal disturbances, salivation, and syncope, which were limitations in earlier studies of xanomeline monotherapy [10]. This synergistic mechanism allows xanomeline-trospium chloride to provide therapeutic efficacy with an optimal safety and tolerability profile [12,13]. Xanomeline-trospium chloride is the first approved medication for schizophrenia in this new therapeutic class. This review aims to integrate the current evidence and assess the potential of xanomeline-trospium chloride as a novel therapeutic approach in the management of schizophrenia.

## Review

Methods

We searched MEDLINE, Cochrane Central Register of Controlled Trials, and ClinicalTrials.gov for studies evaluating the effects of xanomeline-trospium chloride in individuals with schizophrenia-spectrum disorders. To enhance completeness, the search terms were also applied in Google Scholar, supplemented by manual searches to identify any potentially missed studies. In addition, the reference lists of all included articles were systematically reviewed to identify further relevant publications. The following are the search strategies used to retrieve the relevant articles: (random* OR placebo OR RCT OR trial) AND (schizophrenia OR schizoaffective) AND (xanomeline AND trospium) OR KarXT OR "muscarinic agonist" OR "muscarinic receptor agonist" OR "M1 agonist" OR "M4 agonist"). Exclusion criteria were non-human subjects, participants below 18 years of age, articles that only evaluated xanomeline monotherapy without trospium, and those focusing on other muscarinic agonists. A narrative review of the relevant articles was performed.

Clinical pharmacology

Xanomeline as a Muscarinic Cholinergic Receptor Agonist

**Figure 1:**
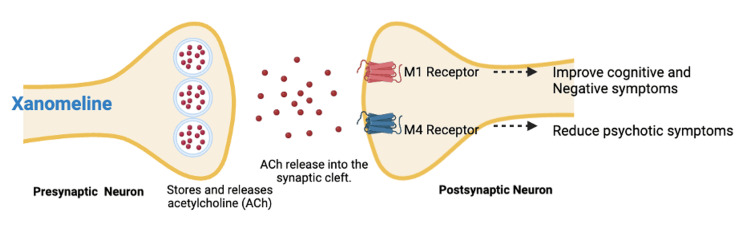
Mechanism of Action of Xanomeline Image credit: Mohsin Raza

Xanomeline, a selective M1 and M4 muscarinic receptor agonist, was first evaluated as a treatment for Alzheimer’s disease and later repurposed for investigation in schizophrenia. Xanomeline-trospium chloride is a novel approach to the management of schizophrenia that targets the cholinergic system, which is involved in cognition, attention, and emotional regulation [14]. The M1 and M4 mAChRs are involved in cognition, negative symptoms, and positive symptoms of schizophrenia (Figure 1) [15,16]. These receptor subtypes are important in regulating the activity of other neurotransmitter systems, such as the dopamine and glutamate systems, which have been implicated in the pathogenesis of schizophrenia [4,10]. Xanomeline-trospium chloride's mechanism of action is therefore believed to offer a better therapeutic approach to the management of this complex disorder. Acetylcholine release from the presynaptic neuron enables these effects on postsynaptic receptors [10,13]. 

Preclinical studies have demonstrated that activation of M4 receptors modulates psychotic and behavioral disturbances, whereas M1 receptor activation is primarily associated with improvements in negative and cognitive symptoms [12,13]. These findings support the mechanistic rationale for the therapeutic potential of xanomeline in schizophrenia. Xanomeline reaches peak plasma concentrations (Tmax) approximately 2.5 hours after administration, with a maximum plasma concentration of 13.8 ng/mL following a 150-mg dose [13]. The drug exhibits very low oral bioavailability (<1%) due to extensive first-pass metabolism [15]. In animal studies, xanomeline is widely distributed, including penetration into the central nervous system, and is predominantly excreted renally, within 24 hours of administration [13].

Clinical trials, such as EMERGENT-2, showed that xanomeline-trospium is effective in improving positive symptoms (hallucinations and delusions) and negative symptoms (social withdrawal and lack of motivation) of schizophrenia. The combination therapy group had a mean PANSS score reduction of 17.4 points, compared to 5.9 points in the placebo group (p < 0.001) [10]. However, xanomeline as a monotherapy was previously associated with significant peripheral side effects, including nausea, salivation, and bradycardia, necessitating the addition of trospium chloride to enhance its tolerability [10,15].

Trospium Chloride as a Peripheral Muscarinic Receptor Antagonist

**Figure 2:**
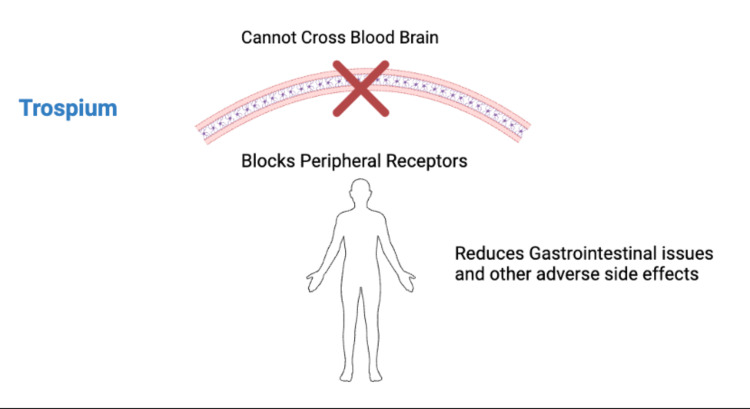
Mechanism of Action of Trospium Image credit: Mohsin Raza

Trospium chloride acts as a peripheral muscarinic receptor antagonist, mitigating xanomeline-induced side effects without crossing the blood-brain barrier (Figure 2). By selectively blocking peripheral receptors, it significantly reduces side effects, like gastrointestinal disturbances and hypersalivation, which were limitations in xanomeline monotherapy [10,15]. Results from the EMERGENT-3 trial showed that this combination reduced the incidence of peripheral side effects compared to xanomeline alone. Additionally, compared to dopamine-blocking antipsychotics, the combination showed lesser metabolic disturbances, minimal weight gain, and fewer extrapyramidal symptoms (EPS), enhancing overall tolerability [10,15].

Absorption and Distribution

Xanomeline-trospium chloride has a biphasic pharmacokinetic profile that is based on the properties of its components. Xanomeline demonstrates rapid absorption, with peak plasma levels reached within one to two hours following oral administration [10]. Trospium is a quaternary amine that exhibits minimal systemic absorption (<10%) and does not cross the blood-brain barrier, hence selectively mitigating peripheral muscarinic side effects while preserving xanomeline’s central actions. This approach may contribute to efficacy, with better tolerability [10,15].

Metabolism and Elimination

Xanomeline is metabolized extensively in the liver via first-pass metabolism, mainly by the cytochrome P450 system, including CYP2D6 and CYP3A4, and has a half-life of seven to nine hours, which supports twice-daily dosing. On the other hand, trospium is mostly excreted in the urine and is eliminated from the body through active renal excretion, with a terminal half-life of about 20 hours [10,15].

Drug Interactions and Implications

The metabolism of xanomeline can be affected by strong CYP inhibitors (e.g., paroxetine) or inducers (e.g., rifampin), which require dose adjustments in these contexts. Also, the use of concomitant anticholinergic drugs may increase the risk of adverse effects, such as dryness of the mouth and constipation. The only problem is that, for trospium, other drugs that compete for renal secretion may increase systemic exposure, and this should be monitored in susceptible populations [15].

Special Populations

Patients with hepatic or renal disease need careful dosage adjustment to prevent adverse effects related to drug accumulation or lack of response. In CYP2D6 poor metabolizers, genetic testing may be used to individualize the dose and decrease the likelihood of overexposure [10]. This comprehensive pharmacokinetic understanding emphasizes the importance of individualized treatment planning to ensure that Cobenfy is both safe and effective for each patient.

Clinical Development and Trials

Xanomeline was initially studied for the treatment of Alzheimer’s disease, particularly targeting cognitive and behavioral symptoms (including agitation and psychosis) due to its M1 and M4 muscarinic receptor agonist activity [17]. Xanomeline-trospium chloride is a new drug developed from previous studies of xanomeline monotherapy, which was effective in improving psychotic symptoms but was limited primarily because of dose-limiting peripheral cholinergic adverse effects that led to poor tolerability and high discontinuation rates. Specifically, gastrointestinal side effects were prominent, including nausea, vomiting, diarrhea, abdominal cramping, and excessive salivation, reflecting stimulation of peripheral muscarinic receptors. The addition of trospium, a peripherally acting muscarinic antagonist, represented a critical advancement in the development program, thereby addressing these tolerability issues while maintaining central therapeutic effects [10]. Initial Phase 1 studies in healthy volunteers showed that the combination was safer than xanomeline monotherapy in causing cholinergic adverse events and that trospium had limited central nervous system penetration [10,13].

The EMERGENT clinical trial program consisted of several Phase 2 and 3 studies, and EMERGENT-1 was a key Phase 2 trial that enrolled 182 adults with schizophrenia. This five-week, randomized, double-blind, placebo-controlled study showed significant improvements in the PANSS total score, with a mean reduction of 17.9 points compared to 5.9 points in the placebo group (p < 0.0001) [18-21]. It also met primary and secondary endpoints, such as PANSS positive and negative subscale scores, as well as the CGI-S scale [20,21]. Following these encouraging findings, the Phase 3 EMERGENT-2 and EMERGENT-3 trials included larger cohorts of patients (N = 246 and N = 368, respectively) and extended the treatment period. These studies also showed efficacy across multiple symptom dimensions, with EMERGENT-2 having a 9.6-point lower PANSS total score than placebo (p < 0.0001) at week 5 [19-21]. The EMERGENT-3 trial further confirmed these results, showing continued improvement in positive and negative symptoms over 12 weeks [10,21]. The safety profile was favorable across all clinical trials, and most adverse events were mild to moderate and occurred early during treatment. The most common side effects were nausea, vomiting, and constipation, which were generally short-lived and could be managed by dose titration [9,10].

Safety and Tolerability

In the clinical trials of xanomeline-trospium chloride, good safety and tolerability were seen, and most of the side effects were mild to moderate and mostly transient. The most common side effects were nausea, vomiting, and constipation, which were attributed to the cholinergic action of xanomeline [9,10]. Such effects were reduced using trospium and dose titration to improve patient compliance [10,13]. The rates of discontinuation due to adverse events were low (7% in the xanomeline-trospium group versus 6% in the placebo group), suggesting good tolerability [10,13].

In contrast to dopamine receptor antagonists, xanomeline-trospium showed no significant weight gain, metabolic disturbances, or EPS. It had minimal effects on metabolic parameters, including lipid profiles and blood glucose, which is an important advantage for long-term treatment [10]. Also, xanomeline-trospium improved cognitive function in the Cogstate Brief Battery (CBB) in EMERGENT-1 (Phase 2) and the Cambridge Neuropsychological Test Automated Battery (CANTAB) in EMERGENT-2 and EMERGENT-3 (Phase 3), an important benefit that is usually lacking with conventional antipsychotics [19-21].

Severe adverse events, including syncope and urinary retention, were infrequent and typically seen in patients with underlying medical conditions, thus necessitating patient monitoring. Long-term safety data show that there is no change in tolerability and that no new safety concerns are identified with prolonged treatment [13]. These findings suggest that xanomeline-trospium is a well-tolerated alternative to available antipsychotic drugs for patients with schizophrenia.

Long-term results from two Phase III trials presented at the 2024 Psych Congress showed that Cobenfy maintained efficacy over 52 weeks in adult patients with schizophrenia, with significant symptom improvement, better quality of life, and minimal adverse effects [20].

Discussion and future perspectives

Xanomeline-trospium chloride demonstrated significant efficacy in reducing overall psychopathology, positive, and negative symptoms, while showing no significant increase in all-cause discontinuation or serious adverse events relative to placebo [22]. Because xanomeline-trospium chloride works through a novel M1/M4 muscarinic pathway rather than dopamine D2 or histamine H1 blockade, it was not linked to many of the side effects that commonly limit antipsychotic treatment. In particular, patients did not experience increased neuromotor symptoms (such as Parkinsonism, akathisia, or dyskinesia), prolactin-related or sexual adverse effects, sedation, insomnia, metabolic disturbances, or weight gain, an outcome against which the drug appeared to be protective. By contrast, gastrointestinal and autonomic effects related to cholinergic activity, including nausea, vomiting, constipation, dyspepsia, and dry mouth, occurred more frequently. Reassuringly, at least in inpatient settings, these effects were typically mild to moderate, short-lived, and did not lead to higher rates of treatment discontinuation.

Xanomeline-trospium chloride may represent a useful treatment option for both acutely ill and stabilized individuals with schizophrenia, particularly for patients who are susceptible to weight gain, metabolic complications, neuromotor adverse effects, prolactin elevation, or sexual dysfunction, as well as the activating or sedating effects commonly associated with postsynaptic dopamine-blocking antipsychotics [22]. Treatment selection may also reasonably incorporate patient preference, especially when tolerability is a priority. Furthermore, given its distinct mechanism of action and potential modulation of presynaptic dopaminergic dysfunction, xanomeline-trospium chloride may offer benefit for patients with persistent positive symptoms or for those who have not achieved adequate response with conventional antipsychotic therapies [23-25]. Patients with acute schizophrenia and cognitive impairments demonstrated significant improvement with xanomeline/trospium compared with placebo [26].

The approval of xanomeline-trospium represents a shift in the management of schizophrenia, but its potential is still to be fully explored. Future work should also examine its utility in more realistic conditions since the patients enrolled in such studies are not necessarily representative of those found in the real world. For example, it is important to determine whether early intervention with muscarinic receptor-targeted therapies can influence the course of the illness in the context of early-phase schizophrenia. This is particularly important because most of the current trials have been conducted on chronically hospitalized patients who may have already developed receptor insensitivity due to the use of conventional antipsychotics.

Another exciting direction is the exploration of the use of xanomeline-trospium in treatment-resistant schizophrenia. Although the drug is effective in the treatment of the general schizophrenia population, its efficacy as an alternative or additional treatment for patients with dopaminergic antagonist-resistant schizophrenia is not fully understood. Current data show that combination therapies with xanomeline-trospium and clozapine or other antipsychotics may have better control of cognitive and psychotic symptoms, with fewer side effects, such as gastrointestinal hypomotility [13].

Beyond schizophrenia, the mechanism of action of xanomeline-trospium can be used in other neuropsychiatric disorders, such as Alzheimer’s disease, where the primary goal is memory and cognition improvement. Its muscarinic receptor modulation may also be helpful in mood disorders and other disorders characterized by neurotransmitter imbalance. These aspects continue to be investigated to further clarify the role of xanomeline-trospium within emerging mechanism-based treatment approaches in psychiatry [20].

## Conclusions

Xanomeline-trospium chloride represents a novel therapeutic approach for schizophrenia through selective muscarinic receptor modulation, demonstrating efficacy across positive, negative, and cognitive symptom domains while minimizing dopaminergic adverse effects. It has a favorable impact on symptom severity, cognition, and tolerability, positioning it as a promising alternative to traditional antipsychotics. However, further investigation is needed to establish its long-term safety, real-world effectiveness, and role in treatment-resistant and early-stage schizophrenia. Continued research into combination strategies and broader neuropsychiatric applications may further define its clinical utility, as well as its potential to advance personalized treatment paradigms in psychiatry.
